# 手术前后血清LDH浓度与大细胞神经内分泌肺癌患者手术预后的相关性分析

**DOI:** 10.3779/j.issn.1009-3419.2021.103.01

**Published:** 2021-05-20

**Authors:** 浩澄 王, 东凤 单, 娅 董, 雪 杨, 壮 于

**Affiliations:** 266003 青岛，青岛大学附属医院肿瘤科 Department of Oncology, The Affiliated Hospital of Qingdao University, Qingdao 266003, China

**Keywords:** 大细胞神经内分泌肺癌, 乳酸脱氢酶, 预后, Lung large cell neuroendocrine carcinoma, Lactate dehydrogenase, Prognosis

## Abstract

**背景与目的:**

血清乳酸脱氢酶（lactate dehydrogenase, LDH）浓度升高，会导致小细胞肺癌、肺腺癌患者预后不佳，但其与大细胞神经内分泌肺癌（lung large-cell neuroendocrine carcinoma, L-LCNEC）患者预后的关系并不清楚，本研究旨在探讨L-LCNEC患者术前血清LDH浓度、术后LDH浓度变化趋势对患者术后无病生存期（disease-free survival, DFS）的影响，从而为判断L-LCNEC的临床预后提供新的思路。

**方法:**

本研究共纳入49例L-LCNEC术后患者，通过医疗记录、电话随访获取患者的临床资料。应用受试者操作特征曲线确定术前LDH的最佳临界值。用*Kaplan-Meier*绘制生存曲线。应用*Cox*比例风险模型计算独立预后因素。

**结果:**

术前血清LDH最佳临界值分别为195.5 U/L。生存曲线显示，术前血清LDH高浓度、术后LDH浓度升高的患者术后DFS缩短（*P* < 0.001, *P* < 0.001）。多因素分析显示，术前LDH浓度、术后LDH浓度变化趋势是L-LCNEC患者术后DFS的独立预后因素（*P* < 0.001, *P*=0.037）。

**结论:**

L-LCNEC患者术前LDH浓度及其术后浓度变化趋势是患者DFS的独立预后因素，术前高浓度、术后浓度升高会导致患者DFS缩短，预后差，应及早进行干预治疗。

## Abstract (en)

**Background and objective:**

Studies have shown that elevated serum lactate dehydrogenase (LDH) concentration can lead to poor prognosis in patients with small cell lung cancer and lung adenocarcinoma, but its relationship with the prognosis of patients with lung large-cell neuroendocrine carcinoma (L-LCNEC) is not clear. This study aims to explore the influence of L-LCNEC preoperative serum LDH concentration and postoperative LDH concentration change trend on the disease-free survival (DFS) of patients after surgery, so as to judge the clinical prognosis of L-LCNEC provides new ideas.

**Methods:**

Collected the clinical data. The receiver operating characteristic (ROC) curve was used to determine the optimal cut-off value, while the *Kaplan-Meier* and *Cox* proportional hazard model were used to analyze data.

**Results:**

DFS was shortened in patients with high serum LDH concentration before operation and increased LDH concentration after operation (*P* < 0.001, *P* < 0.001). The preoperative LDH concentration and postoperative LDH concentration change trend were independent prognostic factors for patients (*P* < 0.001, *P*=0.037).

**Conclusion:**

Preoperative LDH concentration and its postoperative concentration change trend in patients with L-LCNEC are independent prognostic factors for DFS of patients.

大细胞神经内分泌肺癌（lung large-cell neuroendocrine carcinoma, L-LCNEC）于1991年由Travis等首次命名^[1]^，根据2015年世界卫生组织（World Health Organization, WHO）的分类标准，将LCNEC归为神经内分泌肿瘤，镜下特点包括肿瘤细胞大，细胞质丰富且多为嗜酸性，多形性明显，核仁明显，高有丝分裂率，常伴有坏死等^[2]^。LCNEC发病率低，在通过手术治疗的的肺癌患者中，L-LCNEC的发病率为2.1%-3.5%^[3]^。L-LCNEC恶性程度高，同时兼有神经内分泌肿瘤形态学分化特征及大细胞肺癌特性，具有较强的侵袭性，治疗效果较差^[4]^，5年生存率在15%-57%之间^[5]^。因此在开始治疗前，如果可以预测患者预后，将对临床工作具有指导意义。

近年来研究^[6-9]^发现，血清乳酸脱氢酶（lactate dehydrogenase, LDH）浓度与不同病理类型的肺癌患者预后相关，LDH水平升高会导致小细胞肺癌、肺腺癌患者预后不佳，但目前并没有研究指出LDH水平与L-LCNEC患者的预后是否相关。本研究旨在探讨手术前血清LDH浓度、手术前后LDH浓度变化趋势与L-LCNEC患者术后病情进展之间的关系，以期发现可以预测患者预后的指标。

## 1 资料与方法

### 1.1 一般资料

本研究为回顾性分析，通过青岛大学附属医院病历系统，收集2014年1月-2019年12月在我院接受手术治疗的L-LCNEC患者。纳入标准：①患者行“肺癌根治术+系统性淋巴结清扫术”，术后标本经病理学确诊为L-LCNEC；②术前完善全身检查，未发现远处转移，术前分期为Ⅰ期-Ⅲa期；③病历资料完整。排除标准：①具有其他恶性肿瘤病史；②术后标本病理类型为复合型癌；③合并肝功能不全的患者（谷丙转氨酶/谷草转氨酶（alanine aminotransferase/aspartate aminotransferase, ALT/AST） > 2.5倍正常值上限）；④合并心肌梗死、脑梗死、肺栓塞等严重基础疾病；⑤临床资料、随访信息不全。本研究已获得青岛大学附属医院伦理委员会批准。根据纳入及排除标准，最终共纳入L-LCNEC术后患者49例。

### 1.2 数据收集与随访

统计患者基本信息，包括性别、年龄、吸烟史、美国东部肿瘤协作组（Eastern Cooperative Oncology Group, ECOG）评分、疾病相关情况（包括肿瘤部位、类型、大小、淋巴结转移情况、术后分期、术前3天内的血清LDH浓度、术后2周内的血清LDH浓度、术后是否行放化疗）。

结局指标为患者的无病生存期（disease-free survival, DFS），定义为自确诊日期至疾病复发日期。随访方式包括查阅电子病历系统、电话随访，随访截止时间为2020年8月31日。

### 1.3 统计学方法

应用SPSS 25.0统计软件进行统计分析，绘制受试者工作特征（receiver operating characteristic, ROC）曲线，用于计算手术前LDH的最佳临界值。使用*Kaplan-Meier*方法绘制生存曲线，并通过对数秩检验进行比较。应用*Cox*比例风险模型寻找影响预后的因素，进行单因素、多因素分析，以明确独立预后因素。以*P* < 0.05为差异具有统计学意义。

## 2 结果

### 2.1 临床特征

本研究纳入49例患者，男性45例（91.84%），女性4例（8.16%）； < 60岁21例（42.86%），≥60岁28例（57.14%）；38例（77.55%）有吸烟史，11例（22.45%）无吸烟史；38例（77.55%）ECOG 0分，11例（22.45%）ECOG 1分；36例（73.47%）肿瘤位于右肺，13例（26.53%）肿瘤位于左肺；38例（77.55%）为周围型肺癌，11例（22.45%）为中央型肺癌；T分期：T1-T4分别为21例（42.86%）、17例（34.69%）、7例（14.29%）、4例（8.16%）；N分期：N0-2分别为29例（59.18%）、14例（28.57%）、6例（12.25%）；术后病理分期：Ⅰa期14例（28.57%），Ib期7例（14.29%）；Ⅱa期2例（4.08%），Ⅱb期14例（28.57%），Ⅲa期12例（24.49%）；25例（51.02%）术后未接受化疗，8例（16.33%）术后接受“Pemetrexed (P)/Taxol (T)/Gemcitabine (G)+铂”方案化疗，16例（32.65%）术后接受“Etoposide (E)/Irinotecan (I)+铂”方案化疗；所有患者中，仅有2例术后行放射治疗，遂本研究未分析放疗对患者预后的影响。34例（69.39%）在随访过程中发现复发或转移，部分患者同时出现复发及转移，其中12例（35.29%）患者出现原发灶复发，26例（76.47%）出现远处转移，转移部位包括淋巴结（颈部、锁骨上、纵隔）16例（47.06%）、肝8例（23.53%）、肾上腺8例（23.53%）、骨6例（17.65%）和脑5例（14.71%）（表 1）。

**表 1:** 患者临床特征 Clinical characteristics of patients

Category	*n* (%)
Gender	
Male	45 (91.84)
Female	4 (8.16)
Age (yr)	
< 60	21 (42.86)
≥60	28 (57.14)
Smoking status	
Yes	38 (77.55)
No	11 (22.45)
ECOG	
0	38 (77.55)
1	11 (22.45)
Tumor location	
Right lung	36 (73.47)
Left lung	13 (26.53)
Tumor type	
Peripheral	38 (77.55)
Central	11 (22.45)
T staging	
T1	21 (42.86)
T2	17 (34.69)
T3	7 (14.29)
T4	4 (8.16)
N staging	
N0	29 (59.18)
N1	14 (28.57)
N2	6 (12.25)
Postoperative staging	
Ⅰ	21 (42.86)
Ⅱ	16 (32.65)
Ⅲa	12 (24.49)
Postoperative chemotherapy	
No	25 (51.02)
P/T/G+platinum	8 (16.33)
E/I+platinum	16 (32.65)
Recurrence and metastasis	
Lymph node metastasis	16 (47.06)
Primary tumor recurrence	12 (35.29)
Liver metastasis	8 (23.53)
Adrenal metastasis	8 (23.53)
Bone metastasis	6 (17.65)
Brain metastasis	5 (14.71)
ECOG: Eastern Cooperative Oncology Group; P: pemetrexed; T: taxol; G: gemcitabine; E: etoposide; I: irinotecan.	

### 2.2 ROC曲线及生存曲线

对患者术后复查影像学结果进行随访，截止到最终随访日期，以是否出现复发或转移为分组标准，将患者分为稳定组与进展组。绘制ROC曲线时，连续变量为术前患者血清LDH浓度，二变量为病情是否进展。通过ROC曲线，可以得到最大约登指数，以此来确定手术前血清LDH水平的最佳临界值。通过计算，手术前血清LDH水平的最佳临界值为195.5 U/L（敏感度：0.697；特异性：0.750），曲线下面积（area under the curve, AUC）为0.749（95%CI: 0.608-0.891, *P*=0.005）（图 1）。

**图 1:**
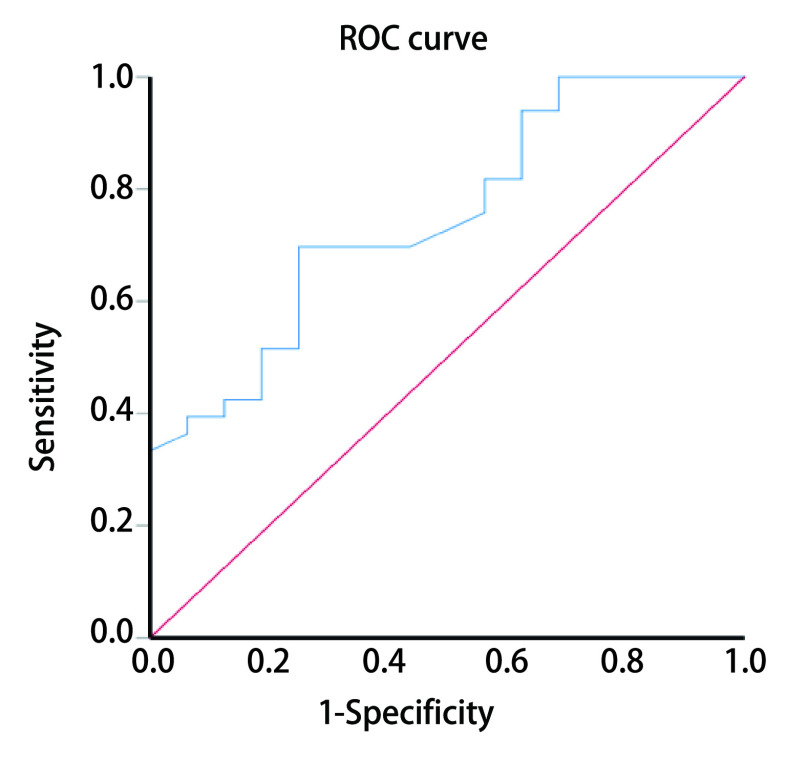
L-LCNEC患者手术前血清LDH浓度的ROC曲线 ROC curve of serum LDH concentration before surgery in L-LCNEC patients. L-LCNEC: lung large-cell neuroendocrine carcinoma; ROC: receiver operating characteristic.

LDH: lactate dehydrogenase.

将195.5 U/L作为临界值，22例患者（44.90%）LDH浓度低于临界值，定义为低LDH组，27例患者（55.10%）LDH水平高于或等于临界值，定义为高LDH组。根据分组，应用对数秩检验进行生存分析，并绘制*Kaplan-Meier*生存曲线图。结果显示，高LDH组（≥195.5 U/L）的生存曲线与低LDH组（< 195.5 U/L）的相比差异具有统计学意义（mDFS：14.07个月*vs* 17.02个月，*P* < 0.001）（图 2）。

**图 2:**
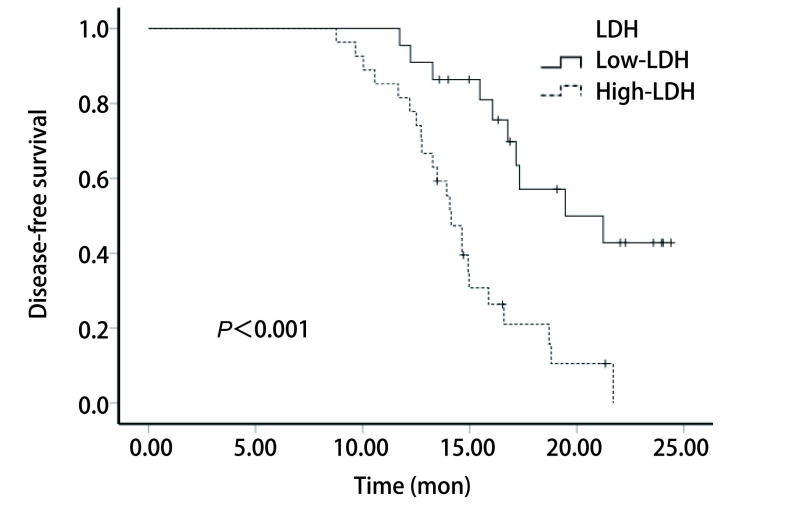
L-LCNEC患者手术前血清LDH水平与患者术后DFS的*Kaplan-Meier*生存曲线 *Kaplan-Meier* survival curve of L-LCNEC patients with preoperative serum LDH levels and postoperative DFS. DFS: disease-free survival.

另外还统计了患者术后2周内的LDH水平，将其与术前浓度相比较，以浓度变化超过5%为有意义，根据浓度变化进行分组，将浓度升高的患者定义为升高组，浓度降低者定义为降低组。经统计，所有患者手术前后LDH浓度变化均超过5%，其中升高组21例（42.86%），降低组28例（57.14%），同样绘制生存曲线图。结果显示，升高组的生存曲线与降低组的相比差异具有统计学意义（mDFS：13.27个月*vs* 17.25个月，*P* < 0.001）（图 3）。

**图 3:**
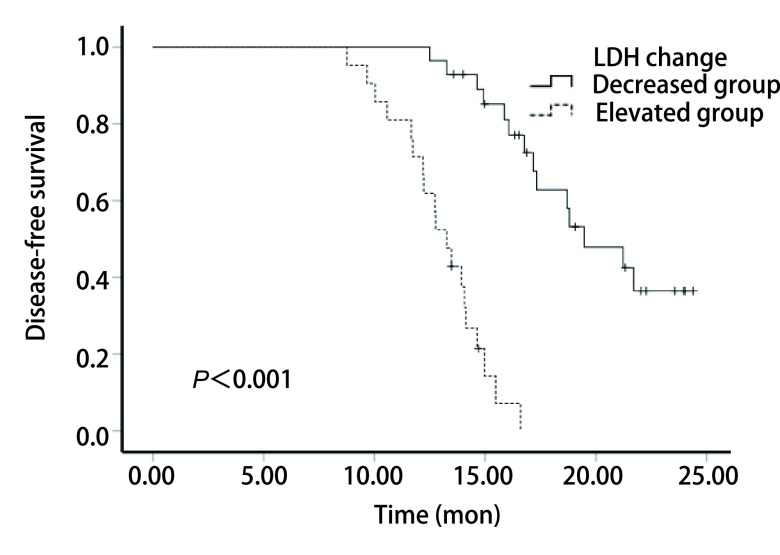
L-LCNEC患者手术前后血清LDH变化与患者术后DFS的*Kaplan-Meier*生存曲线 *Kaplan-Meier* survival curve of L-LCNEC patients' serum LDH changes before and after surgery and patients' postoperative DFS

### 2.3 单因素及多因素分析

对患者的临床资料进行单因素分析，结果表明，与患者DFS相关的因素有吸烟史（*P*=0.028）、ECOG评分（*P*=0.001）、T分期（*P*=0.016）、N分期（*P* < 0.001）、术后分期（*P* < 0.001）、术后化疗（*P*=0.012）、术前LDH浓度（*P* < 0.001）与手术前后LDH变化趋势（*P* < 0.001）（表 2）。

**表 2:** *Cox*单因素分析 *Cox* univariate analysis

Category	HR (95%CI)	*P*
Gender	0.589 (0.177-1.962)	0.389
Age	0.698 (0.352-1.387)	0.305
Smoking status	0.339 (0.129-0.888)	0.028
ECOG	3.713 (1.665-8.280)	0.001
Tumor location	1.056 (0.490-2.274)	0.890
Tumor type	1.536 (0.711-3.320)	0.275
T staging	1.603 (1.093-2.350)	0.016
N staging	4.174 (2.300-7.577)	< 0.001
Postoperative staging	4.296 (2.432-7.588)	< 0.001
Postoperative chemotherapy	0.589 (0.390-0.890)	0.012
LDH concentration before surgery	1.019 (1.012-1.026)	< 0.001
Change of LDH concentration	12.823 (4.759-34.550)	< 0.001
LDH: lactate dehydrogenase.		

将有统计学意义的单因素纳入多因素分析中，结果显示，临床分期（*P*=0.003）、术后化疗（*P* < 0.001）、术前LDH浓度（*P* < 0.001）与手术前后LDH变化趋势（*P*=0.037）是患者术后DFS的独立预后因素（表 3）。

**表 3:** *Cox*多因素分析 *Cox* multivariate analysis

Category	HR (95%CI)	*P*
Smoking status	2.255 (0.649-7.831)	0.201
Tumor location	1.908 (0.725-5.024)	0.191
T staging	2.025 (0.928-4.417)	0.076
N staging	2.633 (0.941-7.365)	0.065
Postoperative staging	8.387 (2.072-33.943)	0.003
Postoperative chemotherapy	0.191 (0.087-0.420)	< 0.001
LDH concentration before surgery	1.023 (1.011-1.036)	< 0.001
Change of LDH concentration	3.430 (1.077-10.922)	0.037

## 3 讨论

L-LCNEC是一种起源于支气管及肺黏膜上皮的神经内分泌细胞的肿瘤^[10]^，1991年，Travis及其同事首次对其命名，将其描述为由大细胞组成的肿瘤，特征包括核质比低、核仁频繁、有丝分裂率高（每10个高倍视野中有10个以上的有丝分裂）和大量坏死^[1]^。2004年WHO将LCNEC归于大细胞癌的一种亚型^[11, 12]^。2015年WHO将LCNEC归于肺神经内分泌癌范畴，诊断标准包括光镜下有神经内分泌形态的存在，高有丝分裂率，大于每10个高倍视野有10个有丝分裂，典型的坏死和至少一种神经内分泌标志物，如突触素、嗜铬粒蛋白A、神经细胞黏附分子CD56/NCAM的免疫组织化学表达阳性^[3, 13]^。L-LCNEC发病率低，根据现有文献^[3]^，在切除的肺癌中，LCNEC的发病率在2.1%-3.5%之间。好发于吸烟男性，多呈周围型肺癌^[14]^，经统计，L-LCNEC手术量占我院肺癌手术总量的1%-2%。由于发病罕见，起病隐匿，侵袭能力强，临床诊断困难，现有的治疗策略多来自于临床试验研究。对于早期患者，手术治疗仍然是首选^[14]^。对于早期可切除的L-LCNEC，Ⅰ期患者5年总生存率为68%-71%，Ⅱ期为32%-89%，Ⅲa期为42%^[15]^，但容易出现远处复发转移^[16]^。因此在开始治疗前，如果可以预测患者预后，将对临床工作具有指导意义。

LDH是糖酵解和糖异生过程中的关键酶。催化丙酮酸和乳酸的相互转化，对能量代谢具有重要意义。LDH、缺氧诱导因子1（hypoxia inducible factor-1, HIF-1）和血管内皮生长因子（vascular endothelial growth factor, VEGF）等因素将肿瘤代谢与血管生成联系在一起，在LDH、HIF-1和VEGF调控作用下，肿瘤细胞迅速生长，使得病情进展^[17-20]^。有研究^[6-9]^表明，LDH水平升高，会导致小细胞肺癌、肺腺癌患者预后不佳。

在本研究中，我们通过ROC曲线，确定手术前LDH浓度的最佳临界值为195.5 U/L，以此为界将患者分为高低两组，绘制生存曲线，结果显示高组患者的中位DFS较低组缩短，差异具有统计学意义（14.07个月*vs* 17.02个月，*P* < 0.001），因此手术前高LDH水平与L-LCNEC患者术后DFS缩短有关。另外，通过比较手术前后LDH浓度变化趋势，我们发现，升高组的生存曲线与降低组的相比差异具有统计学意义（13.27个月*vs* 17.25个月，*P* < 0.001），因此术后LDH水平升高也与L-LCNEC患者术后DFS缩短有关。通过单因素、多因素分析显示，手术前LDH浓度、手术前后LDH变化趋势可能是L-LCNEC患者术后的独立危险因素（*P* < 0.001, *P*=0.037），术前高浓度、术后浓度升高提示术后复发转移出现早，预后差。

这可能对L-LCNEC患者术后治疗提供新思路，是否可以通过降低LDH浓度，使患者生存获益。Yang等^[21]^的研究发现，草酸盐（一种LDH-A抑制剂）能显著抑制NSCLC细胞增殖，对正常肺上皮细胞的毒性要低得多。结果证实针对LDH-A的靶向治疗在NSCLC治疗中的潜在用途。这也进一步支持了靶向LDH在L-LCNEC患者中的治疗潜力。

Iyoda及其同事^[22]^研究发现，与单纯手术相比，术后辅助含铂化疗能显著降低患者的疾病复发率（*P*=0.016, 8）。Sarkaria等^[23]^研究证实，Ib期-Ⅲa期患者术后接受含铂化疗，与单纯手术相比，中位总生存期明显延长（7.4年*vs* 2年）。本研究结果发现，术后化疗也是L-LCNEC患者术后DFS的独立预后因素（*P* < 0.001），这与上述结论相一致，因此术后化疗也是L-LCNEC患者术后重要的预后因素。

我们的研究存在一些局限性。第一，本研究为单中心研究，研究人群比较局限，且样本量较小；第二，本研究为回顾性研究，不能完全排除选择偏倚，信息偏倚；第三，不能完全排除与LDH相关的混杂因素，如饮食、生活习惯等。因此需要进一步开展大规模、多中心的前瞻性研究以证实我们的研究结果。

综上所述，L-LCNEC患者术前LDH浓度及其术后浓度变化是患者DFS的独立预后因素，术前高浓度、术后浓度升高会导致患者DFS缩短，预后差，应及早进行干预治疗。
